# Daratumumab plus VRd in Japanese transplant-ineligible/deferred NDMM patients: Japanese subgroup of the CEPHEUS trial

**DOI:** 10.1007/s12185-026-04185-3

**Published:** 2026-03-27

**Authors:** Kenshi Suzuki, Morio Matsumoto, Hiroyuki Takamatsu, Hiroshi Kosugi, Tadakazu Kondo, Tomoaki Fujisaki, Thierry Facon, Sonja Zweegman, Miku Ito, Chika Sakai, Satoshi Kanai, Tomohiko Nakatogawa, Melissa Rowe, Robin Carson, Saad Z. Usmani

**Affiliations:** 1 Department of Hematology, Japanese Red Cross Medical Center, 4-1-22 Hiroo, Shibuya-ku, Tokyo, 150-8935 Japan; 2 Department of Hematology, NHO Shibukawa Medical Center, Gunma, Japan; 3 Department of Hematology, Kanazawa University Hospital, Ishikawa, Japan; 4 Department of Hematology, Ogaki Municipal Hospital, Gifu, Japan; 5 Department of Hematology, Kobe City Medical Center General Hospital, Hyogo, Japan; 6 Department of Hematology, Matsuyama Red Cross Hospital, Ehime, Japan; 7 Service des Maladies du sang, Hôpital Claude Huriez, Centre Hospitalier Universitaire de Lille, Lille, France; 8 Department of Hematology, Cancer Center Amsterdam, Vrije Universiteit Medical Center, Amsterdam, The Netherlands; 9 Johnson & Johnson, Tokyo, Japan; 10 Johnson & Johnson, High Wycombe, UK; 11 Johnson & Johnson, PA, USA; 12 Memorial Sloan Kettering Cancer Center, New York, NY USA

**Keywords:** Daratumumab, Japan, Newly diagnosed multiple myeloma, Quadruplet therapy, Transplant-ineligible

## Abstract

The phase 3 CEPHEUS trial was conducted in 13 countries starting December 11, 2018 in patients with newly diagnosed multiple myeloma (NDMM) who were transplant-ineligible or for whom transplantation was not planned as initial therapy. Bortezomib, lenalidomide, and dexamethasone (VRd) with daratumumab (D-VRd) provided deeper, more durable responses and lowered the risk of disease progression or death compared with VRd. This analysis evaluated the efficacy and safety of D-VRd specifically in the Japanese subpopulation of the CEPHEUS trial (D-VRd: n = 9; VRd: n = 13). At a median follow-up of 59.0 months, the overall minimal residual disease (MRD) negativity rate was 77.8% with D-VRd and 46.2% with VRd (odds ratio: 4.08 [95% confidence interval: 0.60, 27.65]). The complete response or better rate was 88.9% with D-VRd and 76.9% with VRd. Median progression-free survival was not reached in either group, with a hazard ratio of 0.34 favoring D-VRd. The sustained (≥ 12 months) MRD negativity rate was 55.6% with D-VRd and 38.5% with VRd. Efficacy and safety results were similar to those observed in the global CEPHEUS population, supporting the use of D-VRd in Japanese patients with NDMM.

**Supplementary Information:**

The online version contains supplementary material available at 10.1007/s12185-026-04185-3.

## Introduction

In 2022, multiple myeloma (MM) had an incidence of over 180,000 cases and a mortality of over 120,000 deaths per year worldwide [1]. These figures are predicted to increase in the coming decades [2], necessitating robust and effective therapies.

Daratumumab is a human immunoglobulin (Ig) Gκ monoclonal antibody that targets CD38 and has direct on-tumor and immunomodulatory effects [3–7]. This drug was approved globally for use in combination with various regimens for the treatment of newly diagnosed MM (NDMM) and is widely used [8–15]. In the phase 3 MAIA trial, transplant-ineligible (TIE) patients with NDMM treated with triplet therapy (daratumumab plus lenalidomide and dexamethasone) had significantly improved progression-free survival (PFS) and overall survival (OS) than patients treated with lenalidomide and dexamethasone [8, 9]. As a result, this triplet regimen is widely used as first-line therapy for TIE patients with MM [15].

In the phase 3 PERSEUS trial, transplant-eligible patients with NDMM received either induction and consolidation with quadruplet therapy (daratumumab plus bortezomib, lenalidomide, and dexamethasone [D-VRd]) and maintenance therapy with daratumumab and lenalidomide or induction and consolidation with bortezomib, lenalidomide, and dexamethasone (VRd) and maintenance therapy with lenalidomide [16]. This trial showed that the D-VRd regimen increased the depth of response and significantly improved PFS compared with VRd [16].

The phase 3 CEPHEUS trial was conducted with 395 patients with NDMM who were TIE or for whom transplant was not planned as initial therapy (transplant-deferred) to evaluate the efficacy and safety of D-VRd compared with VRd [17]. Data on the efficacy and safety of the D-VRd regimen in Japanese patients remain limited, however such an analysis provides important insights for clinical decision-making in clinical practice in Japan. The D-VRd regimen provided a deeper and more durable response and lowered risk of disease progression or death than VRd alone, while maintaining a similar safety profile [17]. In this subgroup analysis of the CEPHEUS trial, we evaluated the efficacy and safety of D-VRd in the Japanese subpopulation of patients with NDMM who were either TIE or transplant-deferred.

## Materials and methods

### Study design

The CEPHEUS trial was a randomized, open-label, multicenter, phase 3 trial comparing the D-VRd regimen with the VRd regimen in patients with NDMM and for whom transplant was not planned as initial therapy. This trial was conducted and enrolled patients in 92 centers in 13 countries between 11 December 2018 and 7 October 2019 [17]. Eligible patients were stratified by MM International Staging System (ISS) stage and age/transplant eligibility (< 70 years and TIE, < 70 years and transplant-deferred, and ≥ 70 years), then randomly assigned in a 1:1 ratio to receive either D-VRd or VRd. Here, we report the outcomes of a subgroup analysis in patients from Japan.

The trial was conducted according to the ethical principles that have their origin in the Declaration of Helsinki and that are consistent with Good Clinical Practice and applicable regulatory requirements. The protocol and amendments were reviewed and approved by an Independent Ethics Committee or Institutional Review Board at each participating site. Patients or their legally designated representatives provided their written informed consent. Patient data were limited to those necessary to meet the study objectives and were collected/processed with adequate precautions to ensure confidentiality and compliance with applicable data privacy protection laws and regulations. The CEPHEUS trial was registered on ClinicalTrials.gov under the identifier NCT03652064.

### Patients

The key eligibility criteria were as follows: patients for whom high-dose chemotherapy and stem cell transplant was not planned as initial therapy because of their age (≥ 70 years), or 18–70 years with the presence of comorbidities likely to have a negative impact on the tolerability of high-dose chemotherapy with stem cell therapy (TIE patients), or 18–70 years and with refusal of high-dose chemotherapy with stem cell therapy as the initial treatment (transplant-deferred patients); diagnosis of MM per International Myeloma Working Group criteria [18]; Eastern Cooperative Oncology Group (ECOG) performance status score of 0, 1, or 2 and a frailty index score of < 2 on the Myeloma Geriatric Assessment (thereby including patients aged < 80 years) [19, 20]; measurable disease, as assessed by a central laboratory (including IgG, IgA, IgM, IgD, or IgE MM [serum monoclonal paraprotein level ≥ 1.0 g/dL or urine monoclonal paraprotein level ≥ 200 mg/24 h] or light chain MM without measurable disease in serum or urine [serum Ig free light chain ≥ 10 mg/dL and abnormal serum Ig kappa:lambda free light chain ratio]).

### Treatment

Patients in both groups received the VRd regimen as follows: for cycles 1–8, 21-day cycles of bortezomib as a subcutaneous injection (1.3 mg/m^2^) on days 1, 4, 8, and 11; lenalidomide orally (25 mg) on days 1 to 14; and dexamethasone orally (20 mg) on days 1, 2, 4, 5, 8, 9, 11, and 12 (for patients aged ≥ 75 years or with body mass index < 18.5 kg/m^2^, 20 mg on days 1, 4, 8, and 11). For cycle 9 and beyond, 28-day cycles of lenalidomide on days 1 to 21 and dexamethasone orally (40 mg) on days 1, 8, 15, and 22 were given until disease progression or unacceptable toxicity. Patients allocated to the D-VRd group also received daratumumab 1800 mg subcutaneously once every week in cycles 1 and 2, every 3 weeks in cycles 3–8, and every 4 weeks in cycle 9 and beyond until disease progression or unacceptable toxicity.

Dose modification of daratumumab was not permitted, and dose interruptions or delays were used to manage daratumumab-related toxicities. Bortezomib, lenalidomide, and dexamethasone dose reductions were permitted, and the treatment schedule could be modified for the management of treatment-related toxicities. The pre- and post-administration medications have been published previously [17].

### Endpoints

The primary endpoint was the overall minimal residual disease (MRD) negativity rate. The MRD negativity rate was defined as the proportion of patients who achieved complete response (CR) or better and MRD negative status (at 10^-5^) by bone marrow biopsy/aspirate after randomization, but prior to disease progression, subsequent anti-myeloma therapy, or both. Key secondary endpoints were CR or better rate, PFS, and sustained MRD negativity rate (10^-5^) ≥ 12 months. We also assessed quality of life using the European Organisation for Research and Treatment of Cancer Quality of Life Questionnaire–Core 30 (EORTC QLQ-C30) global health status domain score. Other secondary endpoints have been published previously [17].

Safety evaluations included adverse events (AEs), physical examinations, electrocardiogram monitoring, clinical laboratory parameters, vital sign measurements, and ECOG performance status. All toxicities were graded according to the National Cancer Institute Common Terminology Criteria for Adverse Events version 5 and coded using the Medical Dictionary for Regulatory Activities coding dictionary.

### Statistical methods

The sample size and power calculations and statistical analysis methods for the CEPHEUS trial have been reported previously [17]. The intention-to-treat (ITT) analysis set comprised patients randomly assigned to a treatment group, and the safety analysis set included patients who received at least one dose of any study treatment.

Continuous variables were summarized using descriptive statistics and categorical variables were summarized using frequency tables. The Kaplan–Meier method was used for descriptive summaries of time-to-event endpoints.

The primary endpoint was evaluated in the ITT analysis set, and p-values were calculated using Fisher’s exact test. A Mantel–Haenszel estimate of the odds ratio for stratified or unstratified tables, along with its two-sided 95% confidence interval (CI), was calculated in the global CEPHEUS population and in the Japanese subgroup, respectively. Stratification factors were ISS disease stage (I, II, and III) and age/transplant eligibility (< 70 years and TIE, < 70 years and transplant-deferred, and ≥ 70 years).

For the secondary endpoints, time-to-event efficacy endpoints (including PFS and OS) were assessed, hazard ratios (HRs) and their 95% CIs were estimated using an unstratified Cox regression model with treatment as the explanatory variable for the Japanese subgroup. Binary secondary endpoints, such as CR or better rate, very good partial response or better rate, objective response rate, and the sustained MRD negativity rate, were analyzed in a similar way to the primary endpoint. Treatment-emergent AEs (TEAEs) in the safety analysis set were tabulated overall and by treatment group.

This Japanese subgroup analysis was not powered to detect treatment effect differences between the Japanese and the global CEPHEUS population. Consistency in the efficacy results between the Japanese and the global CEPHEUS population was assessed.

All statistical analyses were conducted using SAS®, version 9.4 (SAS Institute Inc., Cary, NC, USA).

## Results

### Patients

**Fig. 1:**
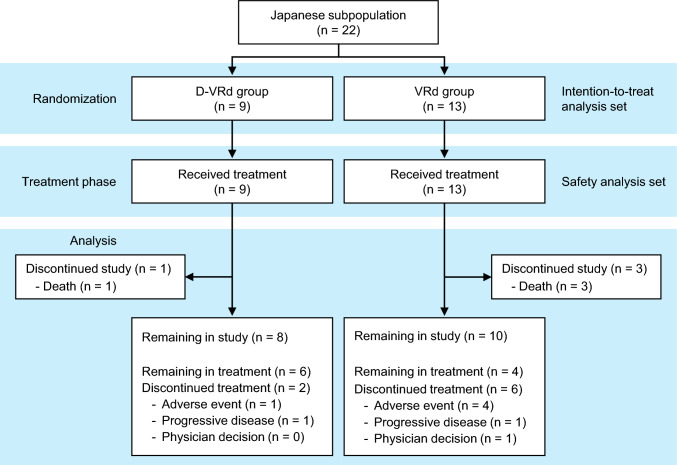
Patient flow. D-VRd, daratumumab plus bortezomib/lenalidomide/dexamethasone; VRd, bortezomib/lenalidomide/dexamethasone

In the Japanese subgroup, nine patients were included in the D-VRd group and 13 patients in the VRd group (Fig. 1). By the clinical cutoff date (7 May 2024), eight patients discontinued treatment (D-VRd group: n = 2; VRd group: n = 6), and four patients discontinued the trial (D-VRd group: n = 1; VRd group: n = 3).

The patient demographics and characteristics at baseline are shown in Table 1. In the Japanese subgroup, the median age was 68.0 years (range: 49–72 years) in the D-VRd group and 74.0 years (range: 61–79 years) in the VRd group. Notably, fewer patients in the D-VRd group were aged ≥ 70 years than in the VRd group (33.3% vs. 84.6%, respectively). Fewer patients in the D-VRd group were male than in the VRd group (two [22.2%] and nine [69.2%], respectively). Four patients (44.4%) in the D-VRd group had an ECOG performance status score of 2, and none of the patients had measurable disease by serum IgA levels. Five patients (55.6%) in the D-VRd group had an ISS disease stage of I and one patient (11.1%) had an ISS disease stage of III. Five patients (38.5%) in the VRd group had an ISS disease stage of I, and four patients (30.8%) had an ISS disease stage of III.

**Table 1:** Patient demographics and characteristics at baseline of the Japanese subgroup and the global CEPHEUS population (intention-to-treat population)

Characteristic	Japanese population		Global CEPHEUS population	
	D-VRd n = 9	VRd n = 13	D-VRd n = 197	VRd n = 198
*Median age, years (range)*	68.0 (49–72)	74.0 (61–79)	70.0 (42–79)	70.0 (31–80)
*Age category, n (%)*				
< 65 years	3 (33.3)	1 (7.7)	36 (18.3)	35 (17.7)
65– < 70 years	3 (33.3)	1 (7.7)	52 (26.4)	53 (26.8)
≥ 70 years	3 (33.3)	11 (84.6)	109 (55.3)	110 (55.6)
*Age/transplant eligibility, n (%)*				
< 70 years and transplant-ineligible	2 (22.2)	0	35 (17.8)	35 (17.7)
< 70 years and transplant-deferred	4 (44.4)	2 (15.4)	53 (26.9)	53 (26.8)
≥ 70 years	3 (33.3)	11 (84.6)	109 (55.3)	110 (55.6)
*Male sex, n (%)*	2 (22.2)	9 (69.2)	87 (44.2)	111 (56.1)
*ECOG-PS, n (%)*				
0	4 (44.4)	7 (53.8)	71 (36.0)	84 (42.4)
1	1 (11.1)	5 (38.5)	103 (52.3)	100 (50.5)
2	4 (44.4)	1 (7.7)	23 (11.7)	14 (7.1)
*Frailty score, n (%)*				
0 (fit)	7 (77.8)	7 (53.8)	124 (62.9)	132 (66.7)
1 (intermediate fitness)	2 (22.2)	6 (46.2)	73 (37.1)	66 (33.3)
*Type of measurable disease, n (%)*				
Detected in serum only	6 (66.7)	7 (53.8)	120 (60.9)	108 (54.5)
IgG	6 (66.7)	5 (38.5)	89 (45.2)	76 (38.4)
IgA	0	2 (15.4)	27 (13.7)	31 (15.7)
Other	0	0	4 (2.0)	1 (0.5)
Detected in serum and urine	1 (11.1)	5 (38.5)	41 (20.8)	45 (22.7)
Detected in urine only	0	1 (7.7)	20 (10.2)	24 (12.1)
Detected in serum free light chains only	2 (22.2)	0	16 (8.1)	21 (10.6)
*ISS disease stage, n (%)*				
I	5 (55.6)	5 (38.5)	68 (34.5)	68 (34.3)
II	3 (33.3)	4 (30.8)	73 (37.1)	75 (37.9)
III	1 (11.1)	4 (30.8)	56 (28.4)	55 (27.8)
*Cytogenetic risk profile, n (%)*				
Standard risk	8 (88.9)	11 (84.6)	149 (75.6)	149 (75.3)
High risk	1 (11.1)	0	25 (12.7)	27 (13.6)
Indeterminate	0	2 (15.4)	23 (11.7)	22 (11.1)
*Median time since multiple myeloma diagnosis, months (range)*	0.82 (0.4–3.8)	1.71 (0.3–4.9)	1.15 (0.4–5.8)	1.26 (0.3–8.0)

D-VRd, daratumumab plus bortezomib/lenalidomide/dexamethasone; ECOG-PS, Eastern Cooperative Oncology Group Performance Status; Ig, immunoglobulin; ISS, International Staging System; VRd, bortezomib/lenalidomide/dexamethasone

The overall median (range) follow-up duration (ITT set) in the Japanese subgroup was 59.0 months (8.5–61.9 months), and there was no notable difference in follow-up duration between the D-VRd and VRd groups. In the D-VRd and VRd groups, the median (range) durations of treatment (safety analysis set) were 55.7 months (0.5–61.2 months) and 56.1 months (1.8–61.9 months), respectively. Relative dose intensity for each drug is shown in Table 2.

**Table 2:** Relative dose intensity (safety population)

	D-VRd n = 9			VRd n = 13
Bortezomib (mg/m^2^) relative dose intensity, %	90.07 (64.1–104.3)			78.96 (48.9–94.1)
Lenalidomide (mg) relative dose intensity, %	82.52 (2.5–100.0)			76.10 (50.6–113.1)
Dexamethasone (mg) relative dose intensity, %	87.74 (25.7–100.0)			73.93 (45.5–100.0)
Daratumumab (mg) relative dose intensity, %	100.0 (66.7–100.0)			NA
	**Cycle 1–2 ** **(n = 9)**	**Cycle 3–8** ** (n = 8)**	**Cycle ≥ 9 ** **(n = 8)**	
Daratumumab (mg) relative dose intensity, %	100.0 (66.7–100.0)	100.0 (83.3–100.0)	100.0 (98.3–100.0)	NA

Data are median (range). D-VRd, daratumumab plus bortezomib/lenalidomide/dexamethasone; NA, not applicable; VRd, bortezomib/lenalidomide/dexamethasone

### Efficacy

**Fig. 2:**
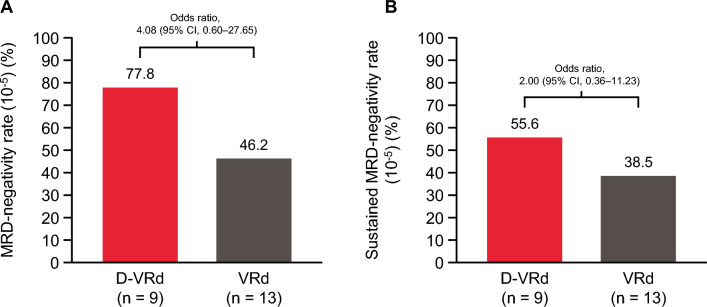
Minimal residual disease negativity rate. Primary endpoint of overall (**A**) MRD negativity rate and the sustained (**B**) MRD negativity rate in the intention-to-treat population. MRD negativity rate was defined as the proportion of patients who achieved complete response or better and MRD-negative status (at 10^−5^) by bone marrow biopsy/aspirate after randomization, but prior to disease progression, subsequent anti-myeloma therapy, or both. Sustained MRD negativity was defined as MRD negativity at two separate examinations at least 12 months apart, without MRD positivity between the examinations. CI, confidence interval; D-VRd, daratumumab plus bortezomib/lenalidomide/dexamethasone; MRD, minimal residual disease; VRd, bortezomib/lenalidomide/dexamethasone

In the Japanese subgroup, the overall MRD negativity rate (10^˗5^) was 77.8% in the D-VRd group and 46.2% in the VRd group (odds ratio: 4.08 [95% CI: 0.60, 27.65]) (Fig. 2A). The cumulative incidence of MRD negativity is shown in Supplementary Fig. 1. The sustained (≥ 12 months) MRD negativity rate (10^˗5^) was 55.6% (95% CI: 21.2%, 86.3%) in the D-VRd group and 38.5% (13.9%, 68.4%) in the VRd group (odds ratio: 2.00 [95% CI: 0.36, 11.23]) (Fig. 2B).

**Fig. 3:**
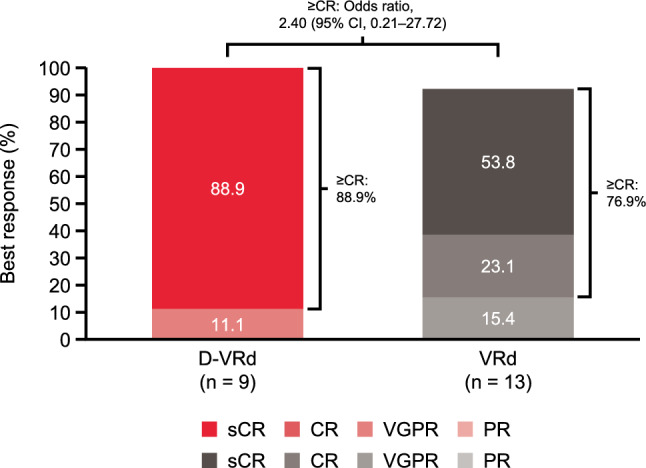
Best response rate. CI, confidence interval; CR, complete response; D-VRd, daratumumab plus bortezomib/lenalidomide/dexamethasone; PR, partial response; sCR, stringent complete response; VGPR, very good partial response; VRd, bortezomib/lenalidomide/dexamethasone

The best confirmed response showed a trend of improvement in the D-VRd group relative to the VRd group (Fig. 3; Supplementary Table 1). Eight patients (88.9% [95% CI: 51.8%, 99.7%]) in the D-VRd group and 10 patients (76.9% [46.2%, 95.0%]) in the VRd group achieved CR or better (odds ratio: 2.40 [95% CI: 0.21, 27.72]). Additional response data are shown in Supplementary Table 1.

**Fig. 4:**
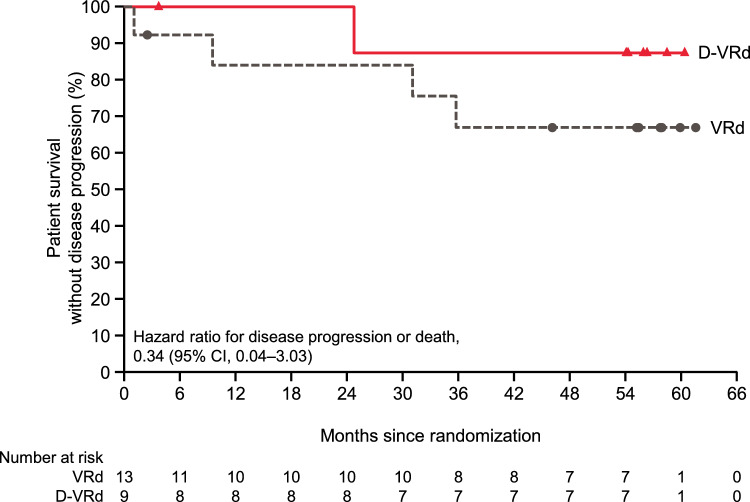
Progression-free survival. CI, confidence interval; D-VRd, daratumumab plus bortezomib/lenalidomide/dexamethasone; VRd, bortezomib/lenalidomide/dexamethasone

The median PFS was not estimable in either the D-VRd or VRd group (95% CI: 24.9 months, not estimable and 9.6 months, not estimable, respectively) (Fig. 4). The D-VRd group had a trend toward improvement in PFS compared with the VRd group (HR: 0.34 [95% CI: 0.04, 3.03]). By the clinical cutoff date, one and three deaths were reported in the D-VRd and VRd groups, respectively. The OS data were immature, and the D-VRd group had a trend toward prolonged OS relative to the VRd group (HR: 0.42 [95% CI: 0.04, 4.07]) (Supplementary Fig. 2). Although the PFS data for the next line of therapy were also immature, the HR favored D-VRd over VRd (HR: 0.46 [95% CI: 0.05, 4.47]) (Supplementary Fig. 3).

Throughout the study, there was no worsening of the EORTC QLQ-C30 global health status domain score with D-VRd compared to VRd (Supplementary Fig. 4).

### Safety

The TEAEs are summarized in Table 3, with the most common TEAEs (defined as those experienced by ≥ 40% of patients based on the sample size) shown in Table 4. All patients experienced at least one TEAE, and Grade 3 or 4 TEAEs were observed in 9 patients (100.0%) in the D-VRd group and 11 patients (84.6%) in the VRd group. The most common TEAEs in the D-VRd group were constipation and insomnia (both 55.6%); lymphopenia, nasopharyngitis, neutropenia, peripheral sensory neuropathy, pneumonia, pyrexia, and oedema peripheral (all 44.4%); cataract, diarrhoea, and thrombocytopenia (all 33.3%); and periodontitis (22.2%). In the VRd group, the most common TEAEs were diarrhoea (84.6%); constipation and peripheral sensory neuropathy (both 76.9%); thrombocytopenia (61.5%); cataract and malaise (both 53.8%); insomnia, nasopharyngitis, and periodontitis (all 46.2%); and neutropenia, oedema peripheral, and pneumonia (all 30.8%). Second primary malignancies were observed in 1 patient (11.1%) in the D-VRd group and 2 patients (15.4%) in the VRd group.

**Table 3:** Summary of safety results (safety population)

	D-VRd n = 9	VRd n = 13
Any TEAE, n (%)	9 (100.0)	13 (100.0)
Grade 3/4	9 (100.0)	11 (84.6)
Any serious TEAE, n (%)	7 (77.8)	12 (92.3)
TEAE leading to discontinuation of bortezomib, n (%)	1 (11.1)	1 (7.7)
TEAE leading to discontinuation of lenalidomide, n (%)	3 (33.3)	4 (30.8)
TEAE leading to discontinuation of dexamethasone, n (%)	1 (11.1)	4 (30.8)
TEAE leading to discontinuation of daratumumab, n (%)	1 (11.1)	NA
TEAE leading to cycle delay or dose modification, n (%)	9 (100.0)	13 (100.0)
TEAE leading to death	0	1 (7.7)
COVID-19, n (%)	3 (33.3)	3 (23.1)
COVID-19-related deaths	0	0

D-VRd, daratumumab plus bortezomib/lenalidomide/dexamethasone; NA, not applicable; TEAE, treatment-emergent adverse event; VRd, bortezomib/lenalidomide/dexamethasone

**Table 4:** Summary of TEAEs with a percentage ≥ 40% in at least one group

	D-VRd n = 9		VRd n = 13	
	Total	Grade 3/4	Total	Grade 3/4
Any TEAE, n (%)	9 (100.0)	9 (100.0)	13 (100.0)	11 (84.6)
Gastrointestinal disorders	9 (100.0)	0	13 (100.0)	3 (23.1)
Constipation	5 (55.6)	0	10 (76.9)	1 (7.7)
Diarrhoea	3 (33.3)	0	11 (84.6)	2 (15.4)
Infections and infestations	9 (100.0)	4 (44.4)	12 (92.3)	5 (38.5)
Nasopharyngitis	4 (44.4)	0	6 (46.2)	0
Pneumonia	4 (44.4)	3 (33.3)	4 (30.8)	2 (15.4)
Periodontitis	2 (22.2)	0	6 (46.2)	1 (7.7)
General disorders and administration site conditions	8 (88.9)	1 (11.1)	8 (61.5)	0
Oedema peripheral	4 (44.4)	0	4 (30.8)	0
Pyrexia	4 (44.4)	0	2 (15.4)	0
Malaise	1 (11.1)	0	7 (53.8)	0
Eye disorders	7 (77.8)	2 (22.2)	8 (61.5)	3 (23.1)
Cataract	3 (33.3)	2 (22.2)	7 (53.8)	3 (23.1)
Blood and lymphatic system disorders	6 (66.7)	5 (55.6)	10 (76.9)	10 (76.9)
Lymphopenia	4 (44.4)	4 (44.4)	2 (15.4)	2 (15.4)
Neutropenia	4 (44.4)	3 (33.3)	4 (30.8)	4 (30.8)
Thrombocytopenia	3 (33.3)	2 (22.2)	8 (61.5)	6 (46.2)
Nervous system disorders	6 (66.7)	1 (11.1)	11 (84.6)	3 (23.1)
Peripheral sensory neuropathy	4 (44.4)	0	10 (76.9)	0
Psychiatric disorders	5 (55.6)	0	8 (61.5)	0
Insomnia	5 (55.6)	0	6 (46.2)	0

D-VRd, daratumumab plus bortezomib/lenalidomide/dexamethasone; TEAE, treatment-emergent adverse event; VRd, bortezomib/lenalidomide/dexamethasone

Seven (77.8%) and 12 patients (92.3%) in the D-VRd and VRd groups, respectively, had serious TEAEs. The most common serious TEAEs were pneumonia (33.3%) and cataract (22.2%) in the D-VRd group, and cataract (23.1%), COVID-19 (15.4%), and pneumonia (15.4%) in the VRd group.

In the VRd group, one patient (7.7%) experienced a TEAE that led to death (malignant lung neoplasm). No patients in the D-VRd group experienced a TEAE that led to death. One patient (11.1%) in the D-VRd group had a Grade 3 or 4 TEAE (pneumonia) and discontinued all study treatment. In the VRd group, four patients (30.8%) had TEAEs that led to treatment discontinuations, of which all were Grade 3 or 4; these were brain stem infarction, peripheral sensorimotor neuropathy, spinal osteoarthritis, cholangiocarcinoma, and lung neoplasm malignant (all n = 1, 7.7%).

All patients experienced TEAEs that led to treatment cycle delays or dose modifications. The most common were COVID-19 and pneumonia (both 33.3%); cataract, erythema multiforme, lymphopenia, neutropenia, peripheral sensory neuropathy, pyrexia, and thrombocytopenia (all 22.2%) in the D-VRd group; and peripheral sensory neuropathy (46.2%), cataract, muscular weakness, and neutropenia (all 30.8%), and diarrhoea, malaise, pneumonia, and thrombocytopenia (all 23.1%) in the VRd group.

Three patients (33.3%) in the D-VRd group and three (23.1%) in the VRd group experienced COVID-19 during the study, but no patients died of COVID-19.

## Discussion

The global CEPHEUS trial demonstrated that the D-VRd regimen significantly increased the depth of response (including MRD negativity and rates of CR or better) and improved PFS, compared with VRd alone, in TIE or transplant-deferred patients with NDMM [17]. Our subgroup analysis demonstrated overall consistency with the global CEPHEUS trial, indicating beneficial effects of D-VRd in the Japanese subgroup. Prior to the CEPHEUS study [17], DRd (from the MAIA study [9]) and D-VMP (from the ALCYONE study [10]) were widely used for TIE NDMM patients in Japan. Based on the results of the CEPHEUS study [17], the D-VRd regimen was approved in Japan in June 2025.

The CEPHEUS study enrolled both TIE patients and transplant-deferred patients, which differs from the previously reported MAIA and ALCYONE studies [9, 10]. This reflects real-world clinical practice, where some patients either decline transplantation or prefer to defer it to later lines of therapy. Furthermore, the CEPHEUS study [17] enrolled patients with a frailty index score of < 2 based on the Myeloma Geriatric Assessment [20], which effectively limited the population to those < 80 years of age. This criterion differs from those used in previous studies [9, 10].

The demographic and baseline characteristics of the Japanese subgroup were generally consistent with those of the overall CEPHEUS population [17], with a few notable differences: fewer male patients and fewer patients with an ECOG performance status score of 1 were included in the Japanese subgroup, and none of the Japanese patients in the D-VRd group had measurable disease by serum IgA levels. Despite these differences, the efficacy of D-VRd in Japanese patients was generally consistent with the global CEPHEUS population [17].

This subgroup analysis showed that the addition of daratumumab to VRd resulted in a higher MRD negativity rate (10^-5^) (77.8% with D-VRd and 46.2% with VRd), and a trend towards improved PFS, with a 66% reduction in the risk of disease progression or death in Japanese patients. Regarding the sustained (≥ 12 months) MRD negativity rate (10^-5^), D-VRd demonstrated a more durable response (55.6% with D-VRd and 38.5% with VRd), supporting the PFS result. Other endpoints, including the percentage of patients who achieved CR or better, also showed similar trends in the Japanese population to those reported in the global CEPHEUS population [17], further supporting the finding of improved outcomes with the addition of daratumumab to VRd.

Regarding safety, the results in the Japanese subgroup were generally consistent with those of the global CEPHEUS population and the known safety profiles of each treatment. The Japanese subgroup had a similar incidence of serious TEAEs in the D-VRd group (77.8%) and a slightly higher incidence in the VRd group (92.3%), relative to the global CEPHEUS population (72.1% in the D-VRd group and 67.2% in the VRd group) [17]. It is possible that the longer duration of VRd treatment in the Japanese subgroup relative to the ITT VRd arm may have contributed to this higher incidence (median duration in the Japanese subgroup: 56.1 months; global CEPHEUS population: 34.3 months [17]). In the Japanese subgroup, more patients in the VRd group than the D-VRd group were aged ≥ 70 years (84.6% vs 33.3%), however, the study was not designed to compare data for patients aged ≥ 70 years. Additionally, the study protocol allowed the patients to discontinue a component of their treatment and continue to receive the other study drugs within their assigned treatment regimen. Therefore, the likelihood of discontinuing all study drugs was, by design, higher in the VRd arm, which involved fewer components than the D-VRd arm, which may have contributed to the higher treatment discontinuation rate due to AEs in the VRd group, as well as the lower relative dose intensity for each drug of the VRd group. These results should be interpreted with caution, especially considering the limited sample size. Deaths due to COVID-19 were observed in both groups in the global CEPHEUS trial, which affected the survival data [17]. In contrast, no deaths due to COVID-19 were observed in either group in the Japanese subgroup.

In Japan, the daratumumab-based regimens for patients with NDMM, such as daratumumab plus lenalidomide and dexamethasone, and daratumumab plus bortezomib, melphalan, and prednisone, have been approved for TIE patients and are recommended in the guidelines [21]. Additionally, findings from other trials have demonstrated the benefit of the D-VRd regimen in patients with NDMM, regardless of transplant eligibility [16, 17]. This subgroup analysis demonstrated the consistency of these results in Japanese patients.

This analysis has some limitations, including the small sample size in the Japanese subgroup.

In conclusion, this trial provided evidence for the efficacy of D-VRd in Japanese patients with TIE or transplant-deferred NDMM. Importantly, the trends were similar to those observed in the global population in the CEPHEUS trial. The addition of daratumumab to VRd therapy clearly demonstrates improved outcomes, supporting the use of daratumumab-based quadruplet regimens as one of the standard-of-care options for Japanese patients with TIE or transplant-deferred NDMM.

## Supplementary Information

Below is the link to the electronic supplementary material.

Supplementary file1 (DOCX 214 KB)

## Data Availability

The data sharing policy of Janssen Pharmaceutical Companies of Johnson & Johnson is available at https://www.janssen.com/clinical-trials/transparency. As noted on this site, requests for access to the study data can be submitted through the Yale Open Data Access [YODA] Project site at http://yoda.yale.edu.
