# Supplementary material for: Daratumumab plus VRd in Japanese transplant-ineligible/deferred NDMM patients: Japanese subgroup of the CEPHEUS trial
Source: Int J Hematol. 2026 Mar 27;124(1):104–13. doi: 10.1007/s12185-026-04185-3 (PMC13319684; doi:10.1007/s12185-026-04185-3)
Supplement: Supplementary file 1 — Supplementary file1 (DOCX 214 KB) [file 12185_2026_4185_MOESM1_ESM.docx]

**Supplementary Materials**

**Daratumumab plus VRd in Japanese transplant-ineligible/deferred NDMM patients: Japanese subgroup of the CEPHEUS trial**

**Author list:**

Kenshi Suzuki^1^, Morio Matsumoto^2^, Hiroyuki Takamatsu^3^, Hiroshi Kosugi^4^, Tadakazu Kondo^5^, Tomoaki Fujisaki^6^, Thierry Facon^7^, Sonja Zweegman^8^, Miku Ito^9^, Chika Sakai^9^, Satoshi Kanai^9^, Tomohiko Nakatogawa^9^, Melissa Rowe^10^, Robin Carson^11^, Saad Z. Usmani^12^

**Affiliations:**

^1^Department of Hematology, Japanese Red Cross Medical Center, Tokyo, Japan

^2^Department of Hematology, NHO Shibukawa Medical Center, Gunma, Japan

^3^Department of Hematology, Kanazawa University Hospital, Ishikawa, Japan

^4^Department of Hematology, Ogaki Municipal Hospital, Gifu, Japan

^5^Department of Hematology, Kobe City Medical Center General Hospital, Hyogo, Japan.

^6^Department of Hematology, Matsuyama Red Cross Hospital, Ehime, Japan

^7^Service des Maladies du sang, Hôpital Claude Huriez, Centre Hospitalier Universitaire de Lille, Lille, France

^8^Department of Hematology, Cancer Center Amsterdam, Vrije Universiteit Medical Center, Amsterdam, The Netherlands

^9^Johnson & Johnson, Tokyo, Japan

^10^Johnson & Johnson, High Wycombe, UK

^11^Johnson & Johnson, PA, USA

^12^Memorial Sloan Kettering Cancer Center, NY, USA


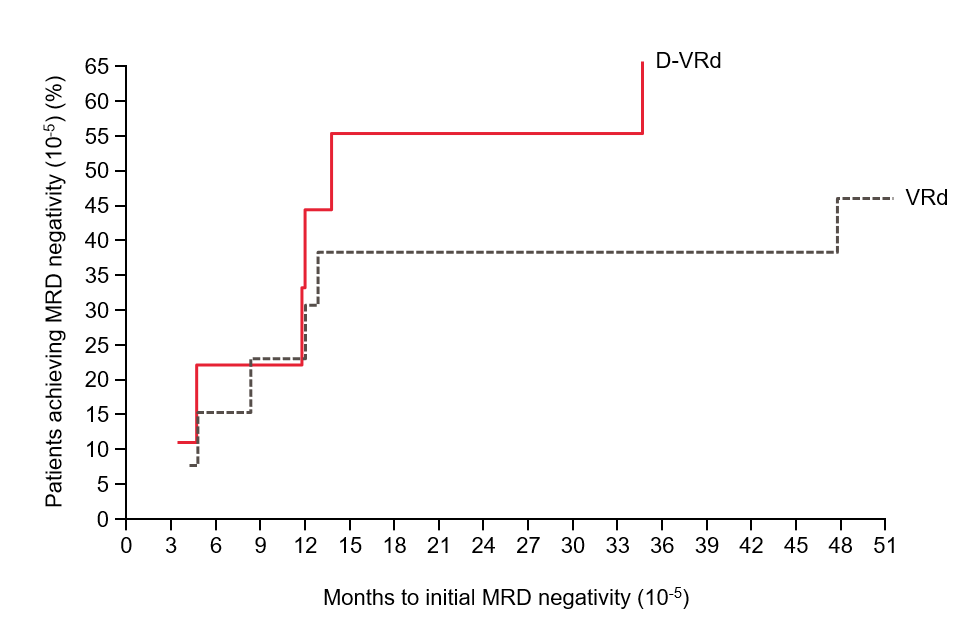


**Supplementary Figure 1.** Cumulative incidence of MRD negativity.

D-VRd, daratumumab plus bortezomib/lenalidomide/dexamethasone; MRD, minimal residual disease; VRd, bortezomib/lenalidomide/dexamethasone.

**
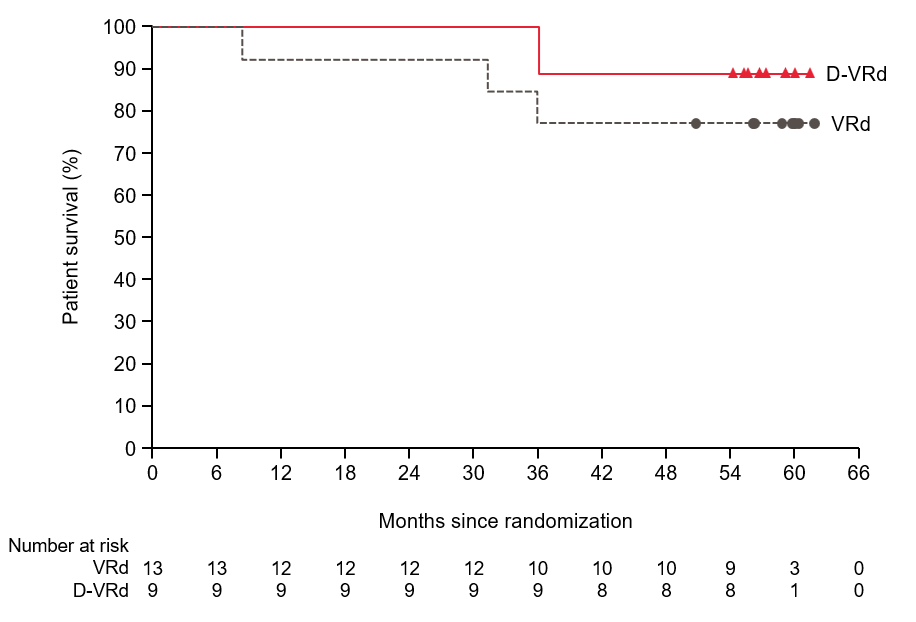
Supplementary Figure 2.** Overall survival.

D-VRd, daratumumab plus bortezomib/lenalidomide/dexamethasone; VRd, bortezomib/lenalidomide/dexamethasone.

**
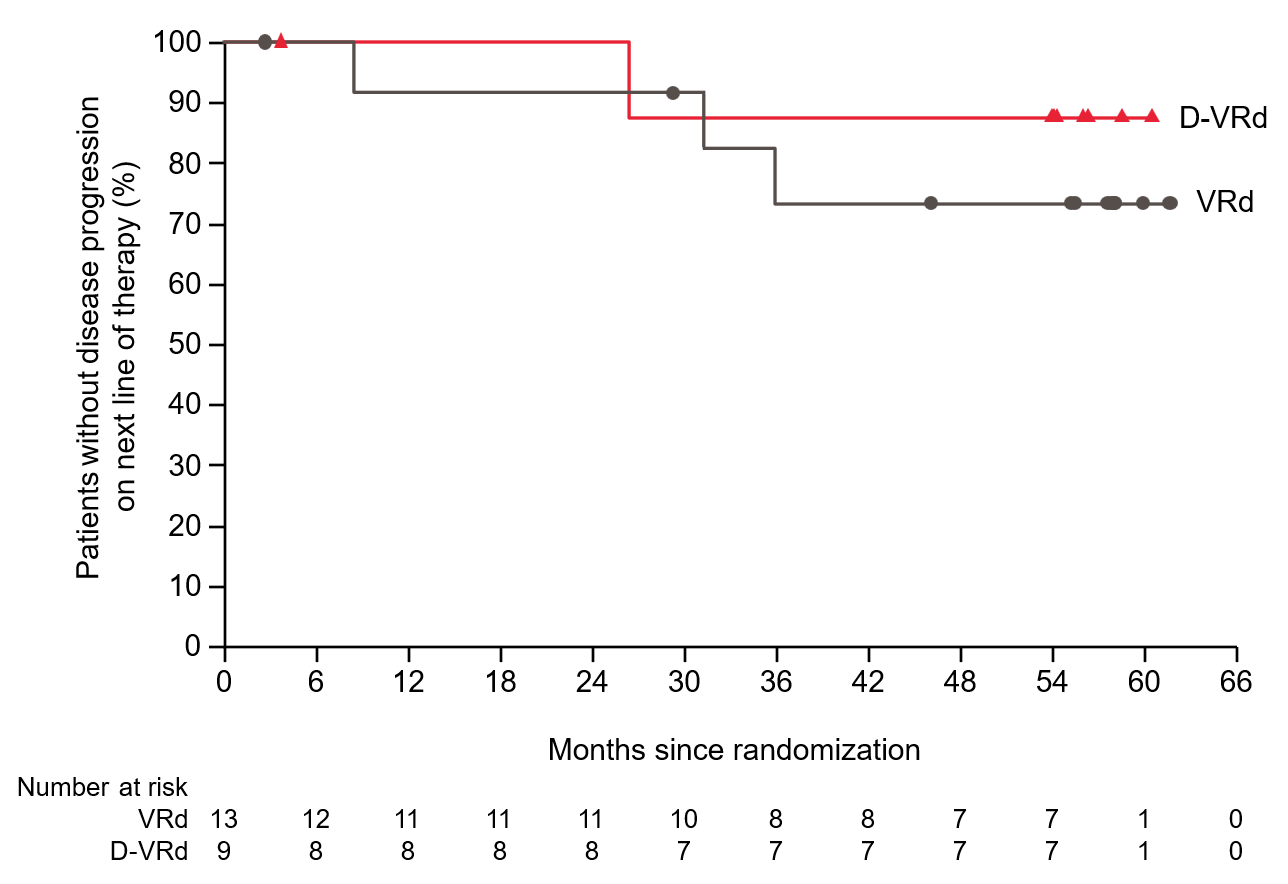
Supplementary Figure 3.** Progression-free survival on next line of therapy.

D-VRd, daratumumab plus bortezomib/lenalidomide/dexamethasone; VRd, bortezomib/lenalidomide/dexamethasone.


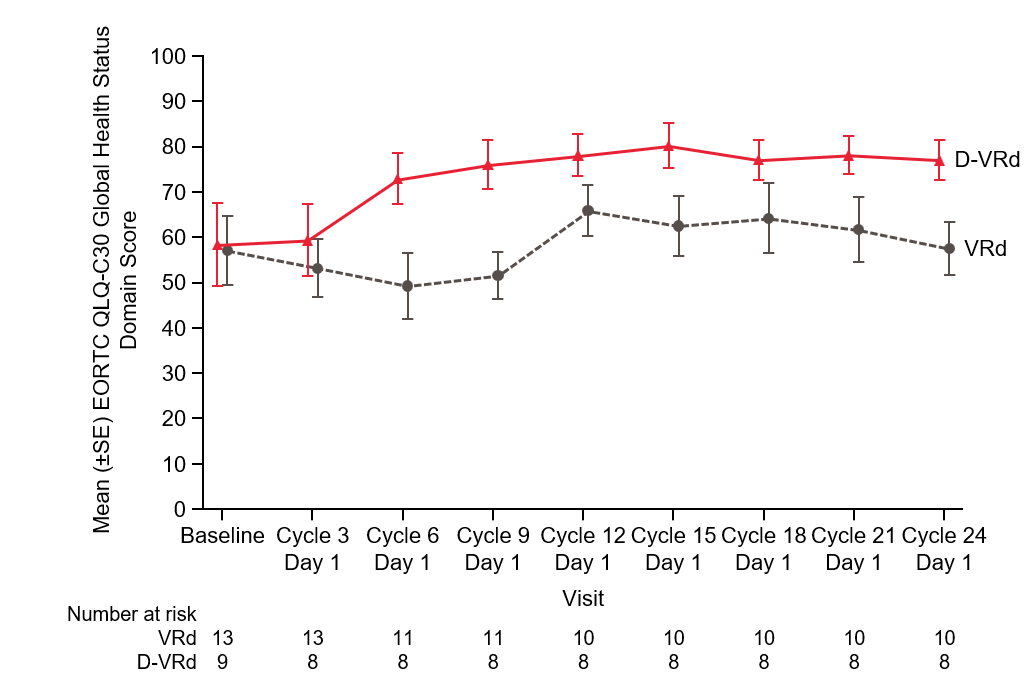


**Supplementary Figure 4.** The EORTC QLQ-C30 global health status domain scores over time.

D-VRd, daratumumab plus bortezomib/lenalidomide/dexamethasone; EORTC QLQ-C30, European Organisation for Research and Treatment of Cancer Quality of Life Questionnaire–Core 30; SE, standard error; VRd, bortezomib/lenalidomide/dexamethasone.

**Supplementary Table 1**. Best confirmed response.

|  | **D-VRd**  **(n = 9)** | | **VRd**  **(n = 13)** | |  |
| --- | --- | --- | --- | --- | --- |
|  | **n (%)** | **95% CI for %** | **n (%)** | **95% CI for %** | **Odds ratio (95% CI)** |
| Stringent complete response | 8 (88.9) | (51.8, 99.7) | 7 (53.8) | (25.1, 80.8) | 6.86 (0.66, 71.72) |
| Complete response | 0 | (NE, NE) | 3 (23.1) | (5.0, 53.8) |  |
| Very good partial response | 1 (11.1) | (0.3, 48.2) | 2 (15.4) | (1.9, 45.4) |  |
| Partial response | 0 | (NE, NE) | 0 | (NE, NE) |  |
| Stable disease | 0 | (NE, NE) | 1 (7.7) | (0.2, 36.0) |  |
| Progressive disease | 0 | (NE, NE) | 0 | (NE, NE) |  |
| Not evaluable | 0 | (NE, NE) | 0 | (NE, NE) |  |
| Overall response^a^ | 9 (100.0) | (66.4, 100.0) | 12 (92.3) | (64.0, 99.8) | NE (NE, NE) |
| Very good partial response or better | 9 (100.0) | (66.4, 100.0) | 12 (92.3) | (64.0, 99.8) | NE (NE, NE) |
| Complete response or better | 8 (88.9) | (51.8, 99.7) | 10 (76.9) | (46.2, 95.0) | 2.40 (0.21, 27.72) |

^a^Including stringent complete response, complete response, very good partial response, and partial response categories.

CI, confidence interval; D-VRd, daratumumab plus bortezomib/lenalidomide/dexamethasone; NE, not estimable; VRd, bortezomib/lenalidomide/dexamethasone.
